# Bitter Taste Receptors TAS2R8 and TAS2R10 Reduce Proton Secretion and Differentially Modulate Cadmium Uptake in Immortalized Human Gastric Cells

**DOI:** 10.3390/ijms26189166

**Published:** 2025-09-19

**Authors:** H. Noreen Orth, Philip Pirkwieser, Maya Giridhar, Valerie Boger, Mark M. Somoza, Andreas Dunkel, Veronika Somoza

**Affiliations:** 1 Graduate School of Life Sciences, Technical University of Munich, 85354 Freising, Germany; n.orth.leibniz-lsb@tum.de; 2 Leibniz-Institute for Food Systems Biology at the Technical University of Munich, 85354 Freising, Germanym.somoza.leibniz-lsb@tum.de (M.M.S.); 3 Chair of Food Chemistry and Molecular Sensory Science, School of Life Sciences, Technical University of Munich, 85354 Freising, Germany; 4 Institute of Inorganic Chemistry, Faculty of Chemistry, University of Vienna, 1090 Vienna, Austria; 5 Institute of Physiological Chemistry, Faculty of Chemistry, University of Vienna, 1090 Vienna, Austria; 6 Chair of Nutritional Systems Biology, School of Life Sciences Weihenstephan, Technical University of Munich, 85354 Freising, Germany

**Keywords:** bitter taste receptors, cadmium, cellular cadmium uptake, metal ion transporters

## Abstract

Beyond sensing bitter-tasting compounds, bitter taste receptors (TAS2Rs) have been demonstrated to play a functional role in proton secretion as a key mechanism of gastric acid secretion (GAS) and the cellular uptake of the zinc metal ion. Given its chemical similarity and comparable effects in GAS, we focused this work on cadmium and hypothesized that gastric TAS2Rs are involved in (i) cadmium-induced inhibition of proton secretion and (ii) in its cellular uptake. To test this hypothesis, immortalized human parietal HGT-1 cells were exposed to 62.5–1000 µM CdCl_2_ for 30 min to elucidate TAS2R-mediated proton secretory activity (PSA) using a fluorescence-based pH cell assay and to quantitate cellular cadmium uptake by ICP-MS. HGT-1 cells exposed to CdCl_2_ exhibited a dose-dependent decrease in PSA, accompanied by a corresponding increase in intracellular cadmium concentrations. Following a *TAS2R* RT-qPCR screening, the functional roles of TAS2R8 and TAS2R10 were clarified using a siRNA knockdown approach, demonstrating that TAS2R8 promotes and TAS2R10 mediates protection against excessive cellular cadmium accumulation. An additional cDNA microarray screening revealed, via gene ontology analysis, a distinct gene association of *TAS2R8* and *TAS2R10* with several metal ion transporters. These results provide the first evidence for a specific role of individual TAS2Rs beyond taste perception, particularly in metal ion homeostasis and gastric physiology.

## 1. Introduction

Bitter taste receptors (TAS2Rs) are part of the chemosensory G-protein coupled receptor (GPCRs) family. While they are primarily known for detecting bitter compounds in the oral cavity, their function extends beyond taste perception. Since their extraoral discovery in transmembrane regions of gastric cells, such as in parietal cells of the stomach [1], TAS2Rs have been implicated in various physiological processes of the stomach, including proton secretory activity (PSA) [1], a key mechanism of gastric acid secretion. Specifically, for receptors TAS2R1, 4, 16, 38, and 43, a functional involvement in PSA has been demonstrated [1,2,3,4]. TAS2Rs also mediate cellular sensing of divalent metal ions, such as Mn^2+^, Mg^2+^, and Fe^2+^ [5,6]. In addition, some of our recent findings show a role for TAS2R43 in regulating zinc homeostasis in human gastric cells in culture [7], thereby giving rise to the hypothesis that TAS2Rs may contribute to protective mechanisms that limit excessive metal ion uptake.

Cadmium (Cd) is a highly toxic heavy metal with no biological function in humans. Its introduction into the human body, primarily via contaminated water, food, or air pollution from industrial activities or tobacco smoke, is linked to severe health consequences, including renal dysfunction [8], carcinogenesis [9], and disruptions in metal homeostasis, reviewed by [10,11]. The World Health Organization (WHO) set, in 2021, a provisional tolerable monthly intake (PTMII) at 25 µg/kg body weight per month [12]. One key factor contributing to Cd’s toxicity is its chemical similarity to the essential micronutrient zinc (Zn) [13]. Notably, both metals have been found to inhibit gastric acid secretion [14,15,16]. The underlying mechanism for zinc remains mainly elusive. Cd, however, affects gastric acid secretion through several mechanisms, including interference with plasma membrane receptors [17,18], disruption of intracellular signaling cascades [19], and inhibition of key enzymes such as carbonic anhydrase and H^+^/K^+^-ATPase [20,21,22]. For zinc, recent findings have identified a functional role for TAS2R43 in mediating zinc-induced inhibition of PSA and reduced cellular uptake [7]. Zn and Cd share similar chemical attributes, particularly a + 2 oxidation state, comparable ionic radius, and similar coordination chemistry. Given this close resemblance, the present study investigated whether TAS2Rs also contribute to the inhibitory effects of Cd on PSA, as well as to the cellular Cd uptake. Cd may enter cells through metal transporters targeting divalent cations, thereby competing with essential metal ions, such as Zn^2+^ [23], Fe^2+^ [24], and Cu^2+^ [25] for binding sites. In particular, members of the Zrt/Irt-like protein (ZIP, SLC39) family and ZnT (SLC30) transporters, which are primarily responsible for maintaining intracellular zinc homeostasis, reviewed by [26], have been shown to facilitate cadmium uptake in mice, for instance via SLC39A8 (ZIP8) [27,28], and SLC39A14 (ZIP14) [28,29]. Intestinal Cd absorption, on the other hand, has been discussed to depend, among other factors such as pH [30], on the presence of competing ions like iron [31,32], zinc [33,34], copper [34], calcium, and phosphorus [35] status in the body. Overall, the average Cd absorption from dietary sources is estimated at about 3–5% [32]. By binding to metallothionein, Cd/it is transported from the intestines to the liver [36] and kidneys [37] of primates, where it accumulates, due to its long biological half-life, for up to 20 years [38]. However, the exact mechanisms underlying Cd transport through the gastric epithelium remain, due to its toxicity, poorly understood. Nevertheless, elucidating these mechanisms is crucial given Cd’s/its harmful effects.

In a recent study using a CRISPR-Cas9 approach [7], we demonstrated that TAS2R43 mediates zinc-induced inhibition of PSA and protects immortalized human parietal cells from excessive zinc uptake. Building on these findings, the present study investigates the role of TAS2Rs in Cd-related effects using a comparable experimental setup in the HGT-1 cell line. Our results reveal that Cd, targeting TAS2R8 and TAS2R10, (i) decreased PSA and (ii) altered cellular cadmium uptake in gastric parietal cells. Notably, TAS2R8 and TAS2R10 differentially regulated cellular Cd uptake. Results from a genome-wide expression profiling using cDNA microarrays supported the Cd-induced functional alterations in TAS2R-specific transport mechanisms. Although Cd transport has been studied widely in the context of essential metal transporters, predominantly in murine models, gene ontology analysis revealed a striking lack of functional associations related to cadmium-specific transport processes. This gap is particularly evident in the human model, highlighting limitations in current functional annotation and understanding.

## 2. Results

The primary hypothesis of this work focused on TAS2Rs regulating PSA as a response to Cd exposure as a key mechanism of gastric acid secretion. Secondarily, it was hypothesized that TAS2Rs are involved in the cellular uptake of Cd.

### 2.1. Cadmium-Induced Reduction of PSA in HGT-1 Cells Is Mediated by TAS2Rs

**Figure 1:**
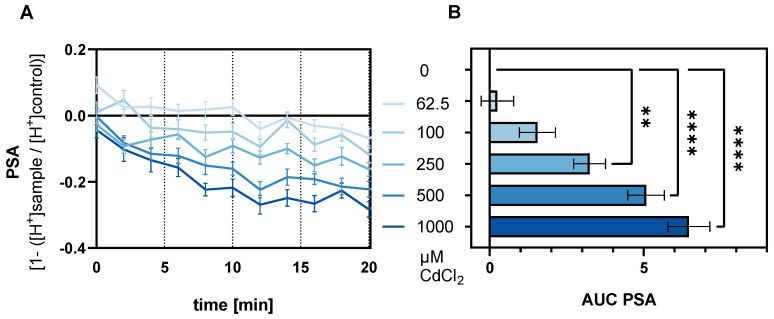
(**A**) Mean PSA of HGT-1 cells treated with CdCl_2_ (62.5–1000 µM) for 30 min and (**B**) Area under the curve of the PSA shown as mean ± SEM analyzed by one-way ANOVA followed by Dunnett’s multiple comparison test with significance displayed as ** = *p* ≤ 0.01. and **** = *p* ≤ 0.0001 with *n* = 4–6; t.r. = 6.

To assess the functional involvement of TAS2Rs in Cd-evoked modulation of mechanisms regulating gastric acid secretion, the PSA was measured using the fluorescent dye SNARF-1AM. The response of cells treated with 62.5, 100, 250, 500, and 1000 µM CdCl_2_ was recorded following a 30 min Cd preincubation over 20 min (Figure 1A) and resulted in areas under the curve (AUC) of 0.251 ± 0.527, 1.550 ± 0.584, 3.244 ± 0.517, 5.077 ± 0.596, and 6.466 ± 0.683, respectively, presented as mean with SEM in Figure 1B. The PSA decreased dose-dependently, observed at 250, 500, and 1000 µM CdCl_2_ compared to the untreated control cells, as also demonstrated by AUC values.

To identify decisive TAS2Rs involved in PSA, mRNA expression levels of human *TAS2Rs* were analyzed in HGT-1 cells following treatment with 100, 500, and 1000 µM CdCl_2_. Gene expression was assessed by RT-qPCR and reported as fold change (FC), calculated as geometric mean. *TAS2R1*, 7, 9, 38, 41, and 60 were excluded from the experiments due to their Ct values over 38.

**Figure 2:**
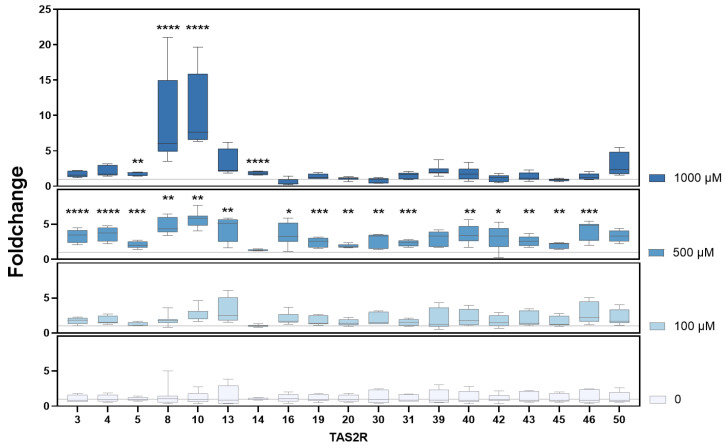
Change of concentration-dependent mRNA expression of 19 bitter taste receptors as fold change (FC) of HGT-1 cells after treatment with CdCl_2_ (100–1000 µM) for 30 min compared to control cells treated with C1 buffer solely (FC = 1.0). Data shown as geometric mean from min to max. *n* = 4–6. t.r. = 3. statistics: Kruskal–Wallis test followed by Dunn’s multiple comparison test: * = *p* ≤ 0.05, ** = *p* ≤ 0.01, *** = *p* ≤ 0.001, **** = *p* ≤ 0.0001.

As shown in Figure 2, 30 min exposure to 500 µM CdCl_2_ resulted in a general upregulation (FC > 1) of *TAS2R* expression compared to untreated control cells (FC = 1), with statistically significant changes (*p* ≤ 0.01) observed for all tested receptor genes, except *TAS2R14*, *39*, and *50*. At 1000 µM CdCl_2_, upregulation was observed only for *TAS2R5*, *TAS2R8*, *TAS2R10*, and *TAS2R14*. A summary of the FC values is provided in Table S1.

**Figure 3:**
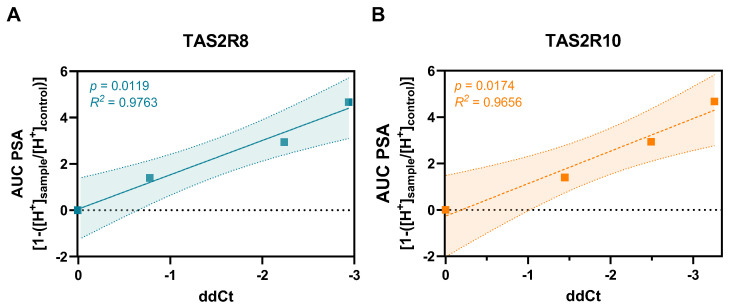
Pearson correlation of AUC PSA and cellular Cd concentration to ddCt of (**A**) *TAS2R8* and (**B**) *TAS2R10* displayed with 95% confidence interval.

Pearson correlation analyses revealed significant associations between the ddCt values of several receptor genes and PSA levels: *TAS2R8* (*p* = 0.0019), *10* (*p* = 0.0320), *14* (*p* = 0.0499), and *16* (*p* = 0.0471). Among these, *TAS2R8* and *TAS2R10* were selected for further investigation due to their strong response effect size (Figure 2) and their highest R^2^ values (Figure 3).

To verify the functional involvement of TAS2R8 and TAS2R10 in the PSA of HGT-1 cells, a transient knockdown (KD) approach using siRNA was employed. KD efficiency was validated by RT-qPCR, showing a mean reduction in gene expression of 69% for *TAS2R8* and 61% for *TAS2R10*.

**Figure 4:**
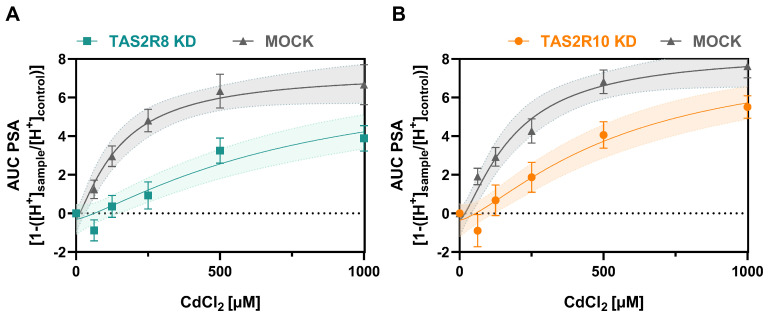
AUC of the PSA in HGT-1 cells with *TAS2R8* (KD) (**A**) and *TAS2R10* (**B**) compared to mock-transfected cells after treatment with CdCl_2_ (62.5–1000 µM).

PSA data following receptor KD were analyzed via one-way ANOVA with Dunnett’s multiple comparison test. Notably, no significant difference was observed between untreated control cells and mock-transfected cells. However, cells with *TAS2R8* or *TAS2R10* knockdown showed a reduced AUC of PSA (*p* ≤ 0.001) compared to MOCK-transfected cells, as illustrated in Figure 4. Detailed results for both KD conditions, *TAS2R8* (Figure 4A) and *TAS2R10* (Figure 4B), are provided in Table S2 and compared to their respective mock controls using an unpaired *t*-test.

These findings support a functional role for TAS2R8 and TAS2R10 in Cd-induced proton secretion mechanisms.

### 2.2. Functional Role of TAS2R8 and TAS2R10 in Cellular Cadmium Uptake

**Figure 5:**
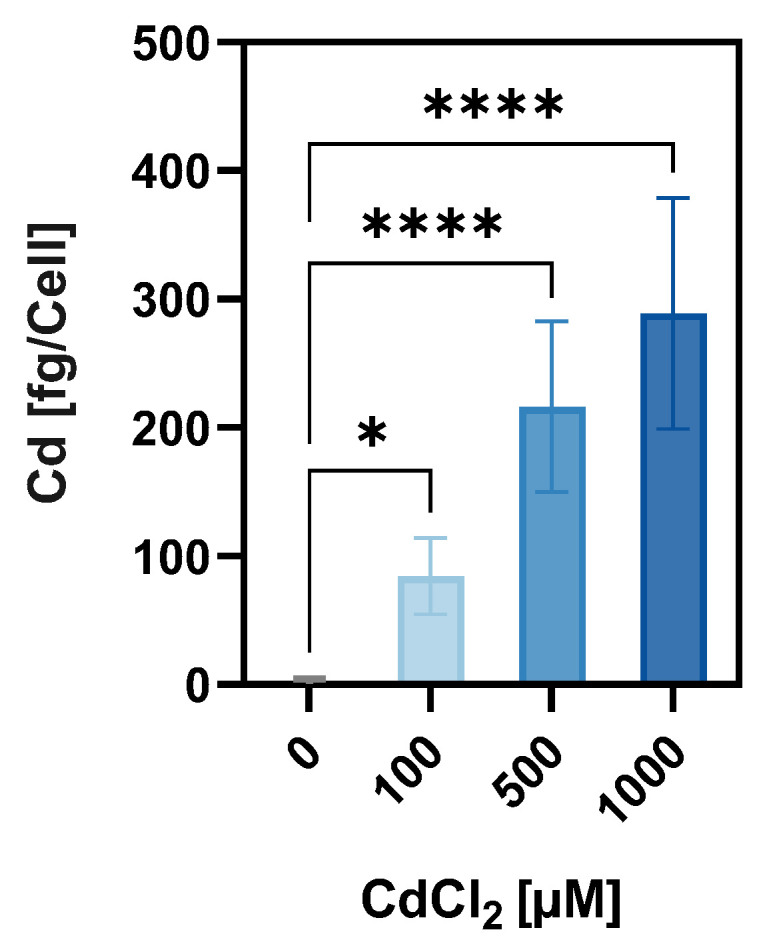
Mean cellular Cd concentration ± SEM in HGT-1 cells after treatment with CdCl_2_ (100–1000 µM) for 30 min after calculation of a nonlinear fit, revealing an IC_50_ of 777.5 µM. The level of statistical significance was calculated via one-way ANOVA and is displayed as * = *p* ≤ 0.05, and **** = *p* ≤ 0.0001.

Given that TAS2Rs have been associated with modulating the uptake of small molecules [39] and metal ions [7], we examined the Cd uptake in HGT-1 cells in response to CdCl_2_ exposure. Cellular Cd^2+^ accumulation was quantified in a bulk experiment using ICP-MS following treatment with 0, 100, 500, and 1000 µM CdCl_2_ for 30 min. As shown in Figure 5, HGT-1 cells displayed a dose-dependent increase in Cd content, with intracellular Cd^2+^ concentrations rising from 1.2 ± 3.1 fg/cell in untreated cells to 84.4 ± 29.4, 216.1 ± 66.4 fg/cell, and 288.9 ± 89.9 fg/cell following exposure to 100, 500, and 1000 µM, respectively. Notably, the efficiency of uptake declined with increasing exposure concentrations, as indicated by the decreasing ratio of administered dose to cellular accumulation: 84% at 100 µM, 43% at 500 µM, and 29% at 1000 µM.

**Figure 6:**
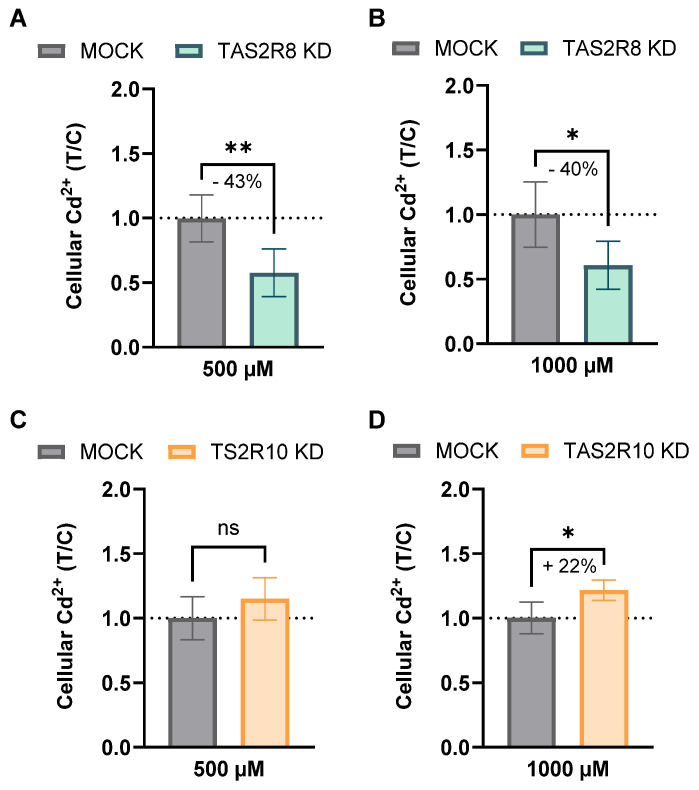
The intracellular Cd^2+^ concentration in *TAS2R8* (**A**,**B**) and *TAS2R10* (**C**,**D**) knockdown HGT-1 cells compared to mock transfected control cells displayed as mean T/C ± SD after treatment with 500 (**A**,**C**) and 1000 (**B**,**D**) µM CdCl_2_ for 30 min and significance after an unpaired *t*-test as follows: * = *p* ≤ 0.05, ** = *p* ≤ 0.01, ns = not significant.

Next, to determine whether TAS2Rs influence Cd uptake directly, intracellular Cd^2+^ levels were quantitated in *TAS2R8* and *TAS2R10* KD cells. In *TAS2R8* KD cells, Cd^2+^ uptake was significantly reduced, by 43% at 500 µM (168.3 ± 53.2) and 40% at 1000 µM (265.1 ± 80.25 fg/cell), compared to mock-transfected controls (292.4 ± 52.6 and 435.0 ± 111.3 fg/cell, respectively), as determined by an unpaired *t*-test. Conversely, *TAS2R10* KD cells exposed to 1000 µM CdCl_2_ showed a 20% increase in Cd^2+^ accumulation (278.0 ± 17.52 fg/cell) relative to mock cells (227.9 ± 27.9 fg/cell). These findings are presented as treatment-over-control (T/C) ratios in Figure 6.

Together, these results support a differential role of TAS2R8 and TAS2R10 in regulating Cd uptake in parietal cells, either by facilitating or limiting intracellular accumulation depending on the receptor subtype.

### 2.3. Genomic Targets of Cellular Cadmium Transport

**Figure 7:**
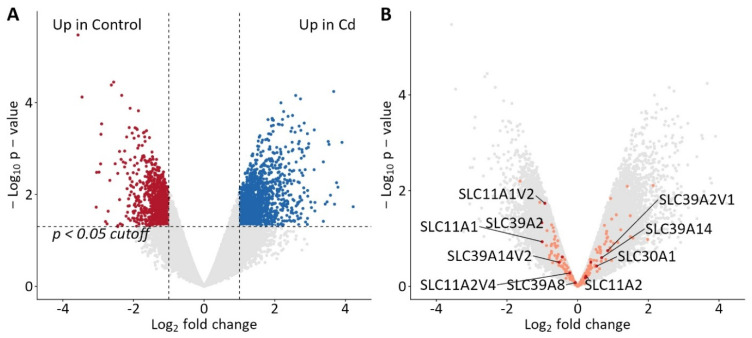
Volcano plot of changes in gene transcripts from a cDNA microarray analysis of wild-type HGT-1 cells upon exposure to CdCl_2_ [500 µM] showing 1572 upregulated (blue) and 963 downregulated (red) transcript variants using cut-off values of ≥±2 for fold change and <0.05 for significance (**A**). Overlay of genes with a gene ontology annotation for Homo sapiens for Cd ion transport (dark red and label) and transition metal ion transport (orange) (**B**).

To gain a broader qualitative understanding of the cellular Cd transport in gastric parietal cells, a cDNA microarray analysis was performed. The aim of this screening was to identify and confirm the relevance of genes known for Cd transport. Therefore, a concentration of 500 µM CdCl_2_ was selected, as most *TAS2Rs* exhibited enhanced gene expression in preceding qPCR experiments. The genome-wide cDNA microarray encompassed 46,957 transcript variants, with results analyzed and visualized using a volcano plot (Figure 7). Applying a threshold of fold change ≥±2 and *p*-value < 0.05, 1572 transcript variants were upregulated and 963 were downregulated in 500 µM Cd-treated versus untreated wild-type HGT-1 cells.

To focus the analysis on genetic targets associated with cadmium ion transport in HGT-1 cells, a gene ontology (GO) [40,41] analysis was performed using the term “cadmium ion transmembrane transport” (GO:0070574), defined as the process by which Cd ions are moved across membranes by transporters or pores. In contrast to corresponding GO terms for other divalent metal ions, only a small number of six genes are currently annotated with this term in Homo sapiens: *SLC11A1*, *SLC11A2*, SLC30A1, *SLC39A14*, *SLC39A2*, and *SLC39A8*. Of these, *SLC11A2* and *SLC39A14* are also annotated with cadmium ion transmembrane transporter activity (GO:0015086). These genes are represented on the cDNA microarray by a total of 10 transcripts and transcript variants (highlighted in dark red in Figure 7B). Upon CdCl_2_ exposure, transcript variant 1 of *SLC39A2*, as well as *SLC39A14*, *SLC30A1*, and *SLC11A2*, showed fold changes of 1.79, 1.60, 1.45, and 1.17, respectively.

However, these differences did not reach statistical significance, albeit with large effect sizes (>0.8). Conversely, *SLC39A2*, *SLC11A1*, *SLC11A1* transcript variant 2, *SLC39A14* transcript variant 2, *SLC11A2* transcript variant 4, and *SLC39A8* were detected at higher levels in control cells compared to Cd-exposed cells, with fold changes of 2.02, 2.00, 1.90, 1.44, 1.17, and 1.05, respectively. Notably, differences in *SLC39A2* (*p* = 0.046) and *SLC11A1* transcript variant 2 (*p* = 0.018) were statistically significant (Table S3). It is important to note that *SLC39A2* variants exhibited the largest fold changes in both up- and down-regulated directions, highlighting the need for comprehensive transcriptomic analyses, verified by functional activity.

Given the limited number of annotated gene targets specifically involved in cadmium transport and the established competition of Cd with other divalent metal transporters, we expanded our gene ontology analysis to include the broader term “transition metal ion transport” (GO:0000041). This category encompasses the transport of transition metals with incomplete d-subshells, including vanadium, manganese, iron, copper, cobalt, nickel, molybdenum, and silver. Using this comprehensive approach, we identified a total of 211 annotated genes, with the majority linked to iron and zinc transport. Among the analyzed transcripts, 12 exhibited significant changes (*p* < 0.05) between control and 500 µM CdCl_2_-treated conditions (Table S4). Several solute carrier transporters—namely *SLC22A17*, *SLC39A2*, *SLC11A1*, *SLC39A10*—as well as *NECTIN 1* showed negative fold changes following CdCl_2_ exposure. In contrast, elevated transcript levels after Cd treatment were observed for genes associated with the regulation of iron ion transport (*HFE*, *FTL*, *TF*, *CYBRD1*), cellular zinc ion uptake and transport (*SLC39A10*, *SLC39A4*).

**Figure 8:**
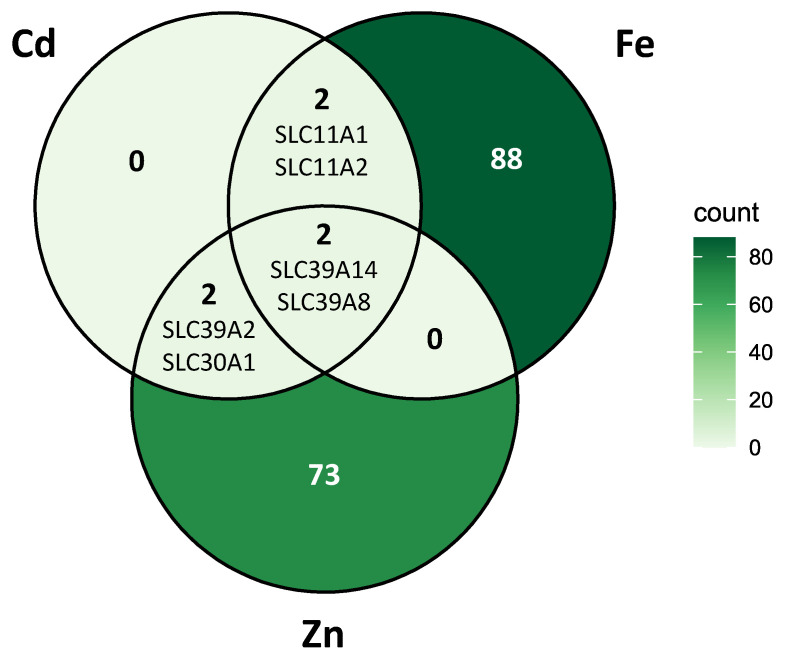
Venn diagram visualizing the qualitative overlap among genes related to the transport of cadmium, iron, and zinc ions.

Since CdCl_2_ exposure led to significant upregulation of several genes involved in the transmembrane transport of iron and zinc ions, we performed a comparative gene ontology analysis to address the sparse annotation of genes exclusively linked to Cd ion transport. Genes annotated under the GO terms for iron ion transport (GO:0006826) and zinc ion transport (GO:0006829) totaled 92 and 77, respectively, clearly illustrating the superior annotation depth for these metals compared to Cd. The qualitative overlap among the gene sets for cadmium, iron, and zinc transport was visualized using a Venn diagram (Figure 8).

This analysis highlights, at the gene level, the apparent uniqueness of iron and zinc ion transport: 88 and 73 genes are uniquely annotated for iron and zinc transport, respectively, whereas each metal shares only four genes with other sets. In contrast, Cd does not possess any uniquely annotated transporter genes; its annotation is entirely based on genes also involved in the transport of other transition metal ions. The overlap between cadmium and iron (*SLC11A1* and *SLC11A2*), as well as cadmium and zinc (*SLC39A2* and *SLC30A1*), consists of two genes each, while two additional genes (*SLC39A14* and *SLC39A8*) are shared among all three elements. Notably, these latter two genes are the only ones jointly annotated for iron and zinc transport. These genes are also referenced for Cd, indicating the lack of any genes encoding iron–zinc transporters that do not also function in Cd transport.

To investigate whether the cellular Cd uptake might be modulated by bitter taste receptor activity, a functional protein interaction network analysis was conducted using the Reactome pathway database [42,43]. Reactome organizes biological processes into functional protein interaction networks, wherein nodes represent proteins and edges signify experimentally validated or computationally inferred interactions relevant to specific biological pathways. The network was constructed using the two taste receptor genes, *TAS2R8* and *TAS2R10*, as input, along with the six genes annotated for cadmium transport in the gene ontology database: *SLC11A1*, *SLC11A2*, *SLC30A1*, *SLC39A14*, *SLC39A2*, and *SLC39A8*. Linker genes connecting these primary nodes were identified using the ReactomeFIViz plugin in Cytoscape [43], and the nature of each interaction was annotated accordingly.

**Figure 9:**
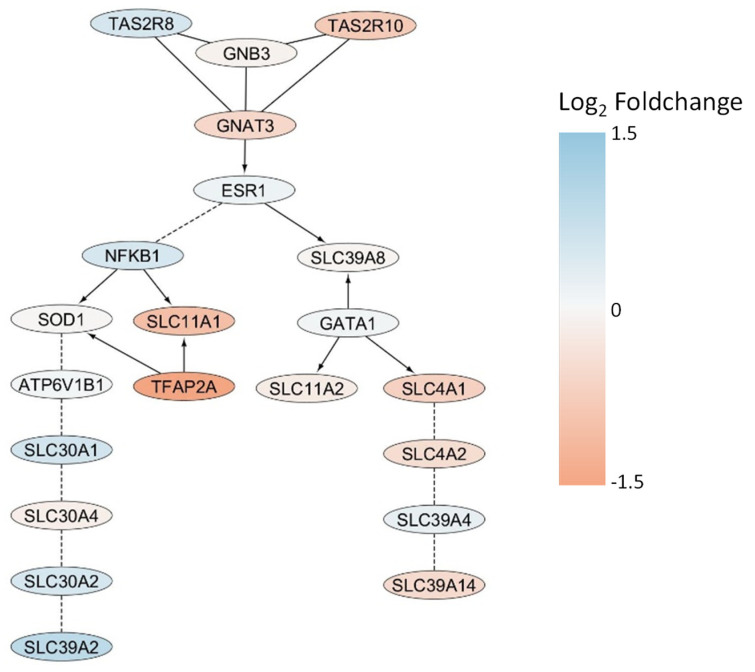
Schematic presentation of the protein interaction network of TAS2R8 and TAS2R10 with metal ion transport genes, colored according to their fold change values from the cDNA microarray analysis.

Figure 9 illustrates the resulting interaction network, with nodes colored according to fold change values from the cDNA microarray analysis. The network consists of three distinct modules interconnected via Estrogen Receptor 1 (*ESR1*). The first module includes both taste receptors (*TAS2R8* and *TAS2R10*) and signal transduction components Guanine Nucleotide-Binding Protein Subunit Beta-3 (*GNB3*) and G Protein Subunit Alpha Transducin 3 (*GNAT3*), which link to *ESR1* as part of the ESR-mediated signaling pathway (R-HSA-8939211). The second subnetwork connects multiple members of the solute carrier protein family 30 (*SLC30A1*, *SLC30A2*, *SLC30A4*) and *SLC39A2* to *ESR1* through Nuclear Factor NF-kappa-B p105 subunit (*NFKB1*) and Superoxide Dismutase 1 (*SOD1*), with *SLC11A1* directly interacting with *NFKB1*. The third module comprises SLC genes from families 39 (*SLC39A4*, *SLC39A14*) and 4 (*SLC4A1*, *SLC4A2*), which are linked to *ESR1* via *SLC39A8* and GATA Binding Protein 1 (*GATA1*).

Integrating the comparative gene ontology analysis with the constructed network reveals that the two genes found at the intersection of zinc and cadmium transport are positioned within the *NFKB1*/*SOD1* branch and exhibit significant upregulation following cadmium exposure. In contrast, genes also associated with iron transport display inverse fold changes and are either directly connected to *NFKB1* or linked through the third branch of the network. By leveraging the comprehensive protein interaction network, including the taste receptor genes, a pathway-based explanation can be proposed for the divergent responses of TAS2R8 and TAS2R10 knockdown cells to Cd exposure. Specifically, *TAS2R8* is upregulated in the presence of Cd, potentially as a result of increased SLC30A2 and *SLC39A2* expression, whereas *TAS2R10*, similar to *SLC4A1*, *SLC4A2*, and *SLC39A14* shows decreased expression under the same conditions. These findings suggest gene-specific regulatory mechanisms in response to Cd exposure, which warrant further validation.

## 3. Discussion

Building on previous research, the current study hypothesized a functional role of TAS2Rs in PSA and Cd uptake mechanisms in human gastric parietal cells. As a result, we identified that Cd exposure led to (i) a dose-dependent decrease in PSA and (ii) differentially regulated cellular Cd uptake.

Our results highlight the dose-dependent decrease in PSA, consistent with previous findings of Cd’s inhibitory effect on gastric acid secretion [14,16], for which the underlying mechanisms are still under discussion. Since TAS2Rs were discovered to co-regulate acid secretion in parietal cells of the stomach [1], their gene expression in HGT-1 cells upon exposure to CdCl_2_ was screened via qPCR (Figure 2). As previously demonstrated [1,7], most *TAS2Rs* were well expressed in HGT-1 cells, except for *TAS2R1*, *7*, *9*, *38*, *41*, and *60*, which were excluded from further analysis due to their Ct values ≥ 38. Most receptors, except *TAS2R14* and *TAS2R50*, responded to the treatment with 500 µM CdCl_2_. *TAS2R8* and *TAS2R10* also exhibited a pronounced fold change when treated with 1000 µM. Both of the latter receptors revealed a concentration-dependent correlation between their respective ddCt values and their PSA (Figure 3). KD experiments of *TAS2R8* and *TAS2R10* resulted in a lower PSA-AUC across all tested CdCl_2_ concentrations (62.5–1000 µM) compared to mock-transfected control cells (Figure 4). PSA values, indicating proton secretion, are plotted as negative values, with more negative values meaning a stronger inhibition of acid secretion (Figure 1). The AUC, however, is calculated as a positive number, summarizing these negative PSA values over time. Hence, a lower AUC describes less negative PSA values, indicating stronger proton secretion and thus, less inhibition. This suggests that knocking down *TAS2R8* or *TAS2R10* reduces Cd’s ability to inhibit acid secretion, supporting their inhibitory role in Cd-induced PSA. These findings are in line with previous observations showing that TAS2R43 can suppress acid secretion in response to ZnCl_2_ [7].

Only a few agonists have been identified for TAS2R8, including chloramphenicol and denatonium benzoate. TAS2R10, on the other hand, appears more promiscuous [44], responding to a broader range of ligands such as chloroquine, and quinine [45], as well as terpenoids and denatonium, with the latter being linked to endothelial barrier protection [46]. While no antagonist has yet been described for TAS2R10, two potent antagonists have recently been identified for TAS2R8, namely S6821 and S7958 [47]. To date, no evidence supports a direct interaction between Cd and TAS2Rs, including TAS2R8 and TAS2R10. Thus, it remains unresolved whether Cd modulates these receptors directly or indirectly via endogenous ligands. The involvement of TAS2Rs in the uptake of small molecules or metal ions, such as resveratrol [39] or Zn^2+^ [7], lends credence to our second hypothesis, which highlights their potential functional role in mediating Cd uptake. We observed a dose-dependent increase in cellular Cd^2+^ accumulation in HGT-1 cells following exposure to CdCl_2_, albeit with decreasing uptake ratios at higher concentrations, possibly caused by saturation (Figure 5). Notably, the KD of *TAS2R8* led to a clear reduction in intracellular Cd^2+^ levels by 43% and 40% at 500 and 1000 µM CdCl_2_, respectively, pointing towards its functional role in cellular Cd uptake (Figure 6). In contrast, *TAS2R10* KD resulted in a 22% increase in Cd^2+^ accumulation at 1000 µM CdCl_2_, thereby indicating a protective effect against excessive cellular Cd accumulation.

The study utilized a comprehensive cDNA microarray screening to characterize the transcriptomic landscape following CdCl_2_ exposure in gastric parietal HGT-1 cells, focusing on the expression and potential regulation of Cd transporters. The use of a CdCl_2_ concentration of 500 µM, established through previous qPCR studies for optimal TAS2R induction, yielded pronounced transcriptome-wide changes: out of nearly 47,000 measured variants, 1572 were significantly upregulated and 963 downregulated, based on conservative fold change and significance thresholds (Figure 7). The following gene ontology analysis was used to center the analysis of the regulated genes on Cd transport, using the GO term “cadmium ion transmembrane transport”. This term is currently sparsely annotated in the human genome, comprising only six genes. Of these, different transcript variants of *SLC39A2*, *SLC11A1*, *SLC39A14*, *SLC30A1*, *SLC11A2* and *SLC39A8* were evaluated for expression changes. While most displayed notable trend-level fold changes, only *SLC39A2* and *SLC11A1* variant 2 reached statistical significance, both being higher in control cells, suggesting repression by Cd. The dual-directionality in *SLC39A2* isoform expression underlines both the complexity of post-transcriptional regulation and the imperative to distinguish among transcript variants in functional studies.

Recognizing (a) the sparse annotation of cadmium transport genes in the human genome, and (b) the competitive uptake of Cd through divalent metal transporters, the GO framework was broadened to “transition metal ion transport,” revealing a total of 211 target genes, predominantly assigned to iron and zinc transport. Notably, eleven solute carrier (SLC) genes, including both presumed and established transition metal transporters (such as *SLC22A17*, *SLC39A2*, *SLC39A10*, *SLC11A1*, and *NECTIN 1*), were significantly downregulated after CdCl_2_ treatment. A contrasting upregulation was observed for several iron- and zinc-associated genes (e.g., *HFE*, *FTL*, *TF*, *CYBRD1* and *SLC39A10*, *SLC39A4*, *TRPM3*).

Future studies using integrated systemic approaches are needed to verify the functional role of carrier proteins and their transcript variants in cellular Cd uptake in response to Cd exposure w/o the presence of competing divalent metal ions. The importance of recognizing the interactions between Cd and other transition metal ions is demonstrated by the subsequent comparative gene ontology and Venn diagram analyses (Figure 8), which confirmed the unique annotation profiles for iron and zinc transporters (88 and 73 uniquely annotated genes, respectively). However, no such exclusivity for Cd-specific genes exists.

Instead, cadmium’s assigned transporter repertoire is nested within the much larger iron and zinc transporter sets, specifically through shared SLC11 and SLC39 proteins. Only four genes overlapped between each pairwise combination, and two genes, *SLC39A14* and *SLC39A8*, both previously reported as Cd-importer [48], were shared among all three cation transport networks. This is strong evidence for the molecular promiscuity underlying Cd uptake: human cells appear to lack specialist machineries for Cd transport, relying entirely on functional redundancy with essential metal pathways.

To elucidate the regulatory and functional connectivity of Cd transport and its potential modulation by bitter taste receptors, a protein interaction network was engineered using Reactome, incorporating both taste receptor (*TAS2R8*, *TAS2R10*) and canonical Cd transporter genes (Figure 9). The resulting network delineated three functional modules, interlinked through *ESR1* (Estrogen Receptor 1), and integrated not only SLC transporters but also key transcriptional and redox regulators *NFKB1* and *SOD1*. The genes shared between zinc and cadmium transport (notably *SLC39A2* [49] and *SLC30A1* [50]) were upregulated and positioned in the *NFKB1*/*SOD1* subnetwork. Furthermore, specific patterning emerged among the taste receptor genes:

Whereas *TAS2R8* was upregulated in tandem with *SLC39A2* transcript variant 1 after CdCl_2_ exposure, its KD revealed a reduction in cellular Cd^2+^ concentrations, thereby indicating a promoting effect of *TAS2R8* and *SLC39A2* transcript variant 1 on cellular Cd transport. In contrast, although *TAS2R10* was also upregulated upon cadmium treatment, KD of this receptor resulted in an increase of intracellular calcium, pointing towards a protective role of TAS2R10 against excessive cellular Cd uptake. This hypothesis is supported by the downregulation of *SLC39A14* transcript variant 2 in response to 500 µM CdCl_2_ exposure. These findings suggest the existence of finely tuned, gene-level regulatory mechanisms governing the differential response of taste receptors and associated metal transporters to Cd, mediated by shared nodes such as *ESR1*, *NFKB1*, and *SOD1* within the broader cell signaling landscape.

Together, these results substantiate the premise that Cd transport in gastric parietal cells does not occur via dedicated, unique molecular machinery. Instead it co-opts the broad specificity and regulatory architecture already in place for essential metal ions such as iron and zinc [51]. The marked absence of uniquely Cd-assigned transporter genes, contrasted with the deep annotation of iron and zinc transporter networks, highlights a significant gap in our molecular understanding and annotation of cellular Cd transport in mammalian systems.

Nevertheless, certain limitations of the present study should be considered: The reliance on transcriptomic readouts may not fully capture regulatory effects occurring at the post-transcriptional or transporter activity levels. The narrow annotation of Cd transporter activity and the low number of unique transporter candidates impede both the discovery and functional dissection of potential Cd-specialized mechanisms. It is also possible that key regulatory responses are cell type-, time-, or concentration-dependent and thus not fully captured at a single concentration or time point.

Future research should address these gaps by extending functional validations such as transporter knockdown or overexpression studies and transporter-specific flux assays, exploring temporal dynamics of transcriptional and translational regulation upon Cd exposure, and leveraging single-cell approaches for greater resolution. Further, systematic improvements to gene ontology databases are warranted to capture noncanonical roles of known transporters in Cd handling. Mechanistic experiments examining the causality between bitter taste receptor signaling and transporter gene regulation, as well as the involvement of central network hubs such as ESR1 and NFKB1, will help clarify the signal integration processes governing cellular Cd responses. Such insights are crucial not only for basic metal biology but also for devising targeted strategies to mitigate Cd toxicity in exposed tissues.

In summary, our study uncovers a novel functional link between PSA and cellular Cd uptake in human gastric cells exposed to CdCl_2_. The distinct effects of *TAS2R8* and *TAS2R10* on Cd uptake suggest differential functional roles that extend beyond gene expression changes and known pathway associations, opening new perspectives for future research. Moreover, these findings challenge the notion of a uniform role for TAS2Rs in cellular Cd uptake and toxicity and highlight the need for deeper receptor-specific mechanistic insights. Notably, only by expanding the gene ontology analysis network to include additional metal ion transporters did potential connections to Cd transport emerge. This underlines the importance of integrative approaches to fully understand Cd transport mechanisms.

## 4. Materials and Methods

### 4.1. Chemicals

Fetal bovine serum (FBS), penicillin-streptomycin (PS), and trypsin/ethylenediaminetetraacetic acid (EDTA) were purchased from PAN Biotech GmbH (Aidenbach, Germany). Gibco Dulbecco’s modified Eagle’s medium Glutamax (DMEM), 1,5-carboxy-seminaphtorhodaflour acetoxymethylester (SNARF-1AM), and nigericin, as well as the reagents for the knockdown, namely SiRNA for TAS2R8 (HSS121406), TAS2R10 (HSS121414), MAPK1 (qHsaCID0006818), Stealth RNAi Negative Control Medium GC Duplex, Opti-MEM serum-reduced medium, and Lipofectamine RNAiMAX were obtained from Thermo Fisher Scientific (Darmstadt, Germany). Dimethyl sulfoxide (DMSO), KCL (≥99.999%), NaCl (≥99.99%), and thiazolyl blue were acquired from Carl Roth (Karlsruhe, Germany). The following were ordered from Merck KGaA (Darmstadt, Germany): 3-(4,5-dimethylthiazol-2-yl)-2,5-diphenyltetrazolium bromide (MTT), 4-(2-hydroxyethyl)-1-piperazine ethanesulfonic acid (HEPES) (≥99.5%), D-glucose (≥99.5%), KOH, and CaCl_2_ (≥99.995%). Cadmium Chloride (CdCl_2_) (≥99.99%) was delivered by Alfa Aesar (Haverhill, MA, USA). Phosphate-buffered saline was obtained from Biozym Scientific GmbH (Hessisch Oldendorf, Germany). As a non-enzymatic cell dissociation solution, the Cellstripper by Corning (Manassas, VA, USA) was used. Propidium iodide (PI) was bought from Miltenyi Biotec (Bergisch-Gladbach, Germany). Calibration and cleaning solutions for the ICP-MS Nexion 5000 were obtained from Perkin Elmer (Rodgau, Germany). Materials for qPCR experiments, including customized plates for TAS2Rs, were acquired from BioRad (Feldkirchen, Germany). C1 buffer composed of 130 mM NaCl, 10 mM HEPES pH 7.4, 5 mM KCl, 2 mM CaCl_2_, and 0.18% glucose.

### 4.2. Cell Culture

Cell culture experiments were performed with the human gastric tumor cell line (HGT-1) obtained from Merck (Merck KGaA, Darmstadt, Germany) in passages between 16 and 36. The cells were cultured in DMEM, supplemented with 10% FBS and 1% PS, under standard conditions at 37 °C and 5% CO_2_. Generally, the cells were treated with CdCl_2_ (62.5–1000 µM) [14] dissolved in C1 buffer under standard conditions for 30 min, based on the retention time of substances in the stomach [52] and harvested with trypsin/EDTA if not declared otherwise [53].

### 4.3. Cell Viability Assay

To exclude cytotoxic effects of the used substances, the metabolic activity of HGT-1 cells was measured for each treatment in the relevant concentration. Therefore, cells were seeded at a density of 50.000 cells/well in transparent 96-well plates for 20–24 h (37 °C, 5% CO_2_). The cells were exposed to solutions of 62.5–1000 µM CdCl_2_ for 30 min before staining under standard conditions for 10 min with 100 µL MTT dye (0.83 mg/mL in DMEM). The evolving formazan was diluted in DMSO and its absorbance was measured with an Infinite M200 plate reader (Tecan, Zurich, Switzerland) at a wavelength of 570 nm and reference 650 nm. The cell viability was calculated by comparing the absorbance of treated with untreated cells in C1 buffer only (100%).

### 4.4. Proton Secretory Activity

PSA is an identifier of bitter and potentially toxic compounds via modulation of proton secretion as a key mechanism of GAS [1]. HGT-1 cells were cultivated at a density of 50.000 cells/well for 20–24 h in black 96-well plates (37 °C, 5% CO_2_), as described previously [7]. The cells were washed with C1 buffer, preincubated for 30 min [52] with CdCl_2_ dissolved in DMEM (62.5–1000 µM) according to the protocol of Orth et al. [7], and washed again before 30 min incubation with 3 µM SNARF-1AM dye in C1 buffer. The dye was aspirated, and the cells were washed again with C1 buffer. For calibration, a potassium buffer (20 mM NaCl, 110 mM KCl, 1 mM CaCl_2_, 1 mM MgSO_4_, 18 mM D-glucose, and 20 mM HEPES) containing 2 µM nigericin was used (pH range 7.0–8.0). Histamine solution (1 mM in C1 buffer) acted as a positive control since it does not target TAS2Rs [54]. Cd-treated cells were covered with C1 buffer before the treatment. The fluorescence emission was monitored by a Flexstation3 (Molecular Devices, San Jose, CA, USA) at wavelengths of 580 and 640 nm after excitation of the dye at a wavelength of 488 nm. Quantitative changes in PSA upon treatment relative to untreated control cells were calculated by subtracting the 580/640 ratio from 1 as reference value of control cells.

### 4.5. Gene Expression

mRNA Expression levels of TAS2Rs in HGT-1 cells were quantitated using real-time-qPCR (RT-qPCR). The cells were seeded at a density of 800,000 in T25 cell culture flasks 20–24 h prior experiment (37 °C, 5% CO_2_). After exposure to CdCl_2_ (100–1000 µM) for 30 min, the cells were lysed using a lysis buffer enriched with 2-mercaptoethanol, and the RNA isolated via a peqGOLD RNA Kit (VWR Peqlab, Radnor, PA, USA) according to the manufacturer’s protocol. The mRNA concentration was measured with a NanoDrop One (A260/280) (Thermo Fisher Scientific, Waltham, MA, USA), gDNA was removed, and cDNA was synthesized using an iScript gDNA Clear cDNA Synthesis Kit (Biorad). By amplifying 50 ng of cDNA with SsoAdvanced Universal SYBR Green Supermix (Biorad) on 25 TAS2Rs [1], RT-qPCR was completed. The cycling conditions were as follows: 60 s/95 °C (activation), 15 s/95 °C (denaturation), 60 s/60 °C (annealing), repeated 44 times. GAPDH and PPIA functioned as reference genes, and data were calculated using the ddCt method, presenting the results as fold change (FC).

### 4.6. Intracellular Cadmium Concentration

The cellular cadmium concentration following CdCl_2_ exposure was quantified in a bulk setup with inductively coupled mass spectrometry (ICP-MS) based on the protocol of Meyer et al. [55] modified for the analysis of Cd^2+^ in HGT-1 cells. The cells were seeded in T25 cell culture flasks 20–24 h before the experiment at a density of 1 × 10^6^ (37 °C, 5% CO_2_). After a washing step with C1 buffer, the cells were exposed to CdCl_2_ (100, 500, and 1000 µM) for 30 min, harvested using the cell stripper solution, and washed again with C1 buffer. A MACSQuant Flow Cytometer (Miltenyi Biotec GmbH) operating at 488 nm gave the cell count with PI, distinguishing dead cells. The cells were microwave digested by adding 1 mL of cell suspension to 4 mL ddH_2_O and 1 mL HNO_3_ (Anton Paar, Courtaboeuf, France) as follows: 20 min ramp to 190 °C, hold for 20 min, and 20 min cool down. After transferring the digests quantitatively into 25 mL volumetric flasks and filling them up to their volume with ddH_2_O, the cadmium content was quantified. Therefore, ^111^Cd was measured using a NexION 5000 ICP-MS operated in MS/MS mode with oxygen in dynamic reaction cell profile (DRC) with ^103^Rh (10 µg/L) as internal standard added automatically by the instrument. After a daily smart tune with NexION setup solution, the instrument parameters were set as follows: ICP-RF power: 1500 W; oxygen flow rate: 1.1 L/min; plasma gas flow: 16 L/min; nebulizer gas flow: 0.88 L/min; auxiliary gas flow rate: 1.2 L/min; RPq = 0.45; dwell time per amu: 50 ms; IGM: focusing. The amount of Cd per cell was calculated with the determined cell number.

### 4.7. Transient Knockdown of TAS2R8 and TAS2R10 Expression

TAS2R8 and TAS2R10 were selected due to the correlation of their ddCt values to the IPX for further investigation on their effect on the studied mechanisms. Their functionality was tested by means of a KD experiment, according to a protocol published by Richter at al. [56]. In order to evaluate the transfection efficiency, Lipofectamine RNAiMAX in Opti-MEM was applied according to the manufacturer’s protocol. The cells were seeded in 24-well plates at a density of 100.000 cells one day prior to the lipofection (37 °C, 5% CO_2_). Two different siRNA sequences were tested for *TAS2R8* (HSS121405 and HSS121406) and *TAS2R10* (HSS121414 and HSS121416). Concentrations of 1, 5, 10, or 50 ng of siRNA for each receptor were added to test a suitable ratio and incubated for 72 h under standard conditions. Stealth RNAi duplex medium GC was used as a negative control, MAPK1 as a positive control, as well as control cells treated only with Opti-MEM. The cells were harvested, RNA isolated, and cDNA synthesized as described above for gene expression analysis, followed by RT-qPCR with the respective pair of primers (SI). The siRNA sequences (HSS121406 with 50 nM for *TAS2R8* and HSS121414 with 10 nM for *TAS2R10*) were chosen due to their most pronounced effect on reducing the receptors’ expression for experiments to determine TAS2R-mediated PSA.

### 4.8. cDNA Microarrays

cDNA microarrays enable a comprehensive screening of gene expression in the human genome. Custom oligonucleotide 60mer gene expression microarrays were conducted as already reported [7,57,58,59]. For this purpose, cells were seeded at a density of 1 × 10^6^ in T25 cell culture flasks for 20–24 h (37 °C, 5% CO_2_) and treated with a concentration of 500 µM CdCl_2_ for 30 min, proceeding with the protocol described by Orth et al. [7]. Briefly, after harvesting the cells, their RNA was isolated as described above, gDNA removed by EZDNase (Invitrogen, Thermo Fisher Scientific, Waltham, MA, USA), and cDNA synthesized by first strand synthesis and reverse transcription using Superscript IV (Invitrogen) and nonamer primers (Tebu Bio), followed by a purification step on QIAquick purification columns (Qiagen, Hilden, Germany). Dried samples were stored until further use at −80 °C for a maximum of 3 weeks. The analysis was conducted after resuspending the cDNA in 12 µL hybridization mix (6 µL MES buffer, 0.7 µL acetylated BSA (10 mg/mL), 0.13 µL herring sperm DNA (10 µg/mL), 0.45 µL QC25-Cy-3 (100 nM), 0.45 µL EcoBioA1 (100 nM), 0.45 µL EcoBioD2 (100 nM) and 3.85 µL DNase free H_2_O) and its hybridization onto human gene expression microarrays for 21 h at 42 °C [60,61,62].

After washing the slides in non-stringent wash buffer (2 min; SSPE; 0.9 M NaCl, 0.06 M phosphate, 6 mM EDTA, and 0.01% Tween20), followed by stringent wash buffer (1 min; 100 mM MES, 0.1 M NaCl, and 0.01% Tween20), and finally for 10 sec in the final wash buffer (0.1 × saline-sodium citrate buffer), the slides were spin-dried using a microarray-centrifuge. The slides were scanned at 532 nm at 2 µM pixel size resolution by an InnoScan1100 microarray scanner (Innopsys, Carbonne, France) with the software Mapix v.8.5.0 (Innopsys, Carbonne, France). Signals were extracted using the NimbleScan 2.6 (NimbleGen Systems Inc., Madison, WI, USA).

Raw data were normalized using the Robust Multichip Average (RMA) [63], and statistical analysis was performed using the limma package version 3.64.3 [64] within the R programming environment version 4.5.1. Venn diagrams were calculated and visualized using the ggVennDiagram package [65], and retrieval of gene ontology information was conducted using the Interface to BioMart databases (R package biomaRt, version 2.64.0) [66]. Network analysis was performed within Cytoscape [67] using the extension app ReactomeFIPlugIn version 8.0.10 [43]. In case of multiple gene transcript variants, the one showing the highest correlation with the expression of the corresponding taste receptor gene was chosen (*TAS2R8*-*SLC39A2*; *TAS2R10*-*SLC39A14*).

### 4.9. Statistical Analysis

Experiments were all conducted with at least three biological replicates (b.r.) and 3–6 technical replicates (t.r.). Statistical analysis was performed after excluding outliers by Nalimov outlier analysis. Normal distribution has been verified by the Shapiro–Wilk test and/or the Kolmogorov–Smirnov test. Statistical significance was evaluated according to one-way ANOVA, Dunnett’s multiple comparison, or Student’s *t*-test for normal distribution and with Kruskal–Wallis test, followed by Dunn’s multiple comparison test or unpaired *t*-test when the assumption of normality is violated, with *p*-values smaller than 0.05, using GraphPad Prism (v.10.6.0). Correlations were calculated by Pearson correlation coefficients. Data were displayed as mean ± SEM. Asterisks indicated the *p* values according to the following scheme: * = *p* ≤ 0.05, ** = *p* ≤ 0.01, *** = *p* ≤ 0.001, **** = *p* ≤ 0.0001. cDNA Microarray data were analyzed with R using a custom annotation and design package as described by Danzer et al. [57].

For the analysis of cDNA microarray data, a custom annotation and design package was developed, comprising a SQLite database that included feature-level information such as x and y array positions and feature set IDs. XYS files generated from NimbleScan 2.1 software (Roche NimbleGen, Madison, WI, USA) were directly imported into R, followed by background correction and normalization using the robust multichip average (RMA) method [63]. Array and preprocessing quality were assessed by inspecting the normalized data from control probes present in the hybridization mix (QC25, ECO1BioA1, ECO1BioD2).

Probe IDs were mapped to the Ensembl database utilizing the R package biomaRt version 2.64.0 [66]. Group differences were visualized using a volcano plot, generated following fold-change calculation (threshold > ±2) and assessment of significance using the limma R package [64].

## 5. Conclusions

This study highlights the functional role of TAS2Rs in regulating proton secretion and Cd uptake in gastric parietal cells. Cd was shown to reduce gastric acid secretion via TAS2R-mediated pathways, while genome-wide analysis revealed additional targets related to metal ion transport. The differential effects of TAS2R8 and TAS2R10 on Cd uptake emphasize the complex interplay between Cd, TAS2Rs, and cellular metal ion transport. This work opens new perspectives for investigating TAS2R-specific interactions and the need to elucidate the specific transport pathways, particularly in the context of essential metal ion homeostasis and human health.

## Data Availability

Data will be made available by the corresponding author on reasonable request.

## Supplementary Materials

The following supporting information can be downloaded at: https://www.mdpi.com/article/10.3390/ijms26189166/s1.
