# Supplementary material for: Bitter Taste Receptors TAS2R8 and TAS2R10 Reduce Proton Secretion and Differentially Modulate Cadmium Uptake in Immortalized Human Gastric Cells
Source: Int J Mol Sci. 2025 Sep 19;26(18):9166. doi: 10.3390/ijms26189166 (PMC12470311; doi:10.3390/ijms26189166)
Supplement: Supplementary file 1 [file ijms-26-09166-s001.zip › ijms-3817555-supplementary.pdf]

Table S1: FC as geometric mean (GM) ± SD on TAS2Rs in HGT-1 cells exposed to $CdCl_2$ against untreated cells (FC = 1) and the p-value after Kruskal-Wallis-Test in followed by Dunn´s multiple comparison test. providing the data for Figure 4.

	**100**			**500**			**1000**		
	GM	SD	p	GM	SD	p	GM	SD	p
TAS2R3	1.67	0.91	0.4516	3.27	0.91	<0.0001	1.60	0.91	0.7196
TAS2R4	1.76	0.91	0.7690	3.57	0.91	<0.0001	2.00	0.81	0.1272
TAS2R5	1.23	0.94	>0.9999	2.02	0.93	0.0002	1.73	0.91	0.0042
TAS2R8	1.71	0.88	>0.9999	4.72	0.93	0.0040	7.67	0.89	<0.0001
TAS2R10	2.73	0.90	0.6576	5.63	0.94	0.0020	9.58	0.85	<0.0001
TAS2R13	2.87	0.85	0.2939	4.00	0.86	0.0076	3.09	0.74	0.1350
TAS2R14	1.01	0.95	>0.9999	1.30	0.97	0.0709	1.80	0.92	<0.0001
TAS2R16	2.00	0.90	0.4407	3.29	0.85	0.0121	0.47	0.82	>0.9999
TAS2R19	1.61	0.90	0.3006	2.41	0.91	0.0008	1.33	0.79	>0.9999
TAS2R20	1.44	0.91	0.4626	1.92	0.96	0.0028	1.04	0.88	>0.9999
TAS2R30	1.81	0.88	0.3260	2.59	0.88	0.0076	0.75	0.71	>0.9999
TAS2R31	1.44	0.91	0.4518	2.31	0.94	0.0001	1.44	0.78	0.5868
TAS2R38	1.83	0.80	>0.9999	4.13	0.84	0.0270	2.37	0.71	0.7733
TAS2R39	1.51	0.79	>0.9999	2.74	0.88	0.0332	2.10	0.75	0.2938
TAS2R40	1.62	0.81	>0.9999	3.42	0.88	0.0018	1.59	0.73	>0.9999
TAS2R42	1.50	0.85	>0.9999	2.50	0.75	0.0251	1.01	0.81	>0.9999
TAS2R43	1.75	0.86	0.3094	2.60	0.92	0.0036	1.24	0.77	>0.9999
TAS2R45	1.49	0.88	0.1957	2.00	0.93	0.0096	0.88	0.81	>0.9999
TAS2R46	2.43	0.84	0.1163	3.89	0.89	0.0006	1.31	0.78	>0.9999
TAS2R50	1.97	0.86	0.7860	3.27	0.93	0.0018	2.70	0.79	0.0310

Table S2: AUC of the PSA in TAS2R8KD and TAS2R10KD cells upon exposure to CdCl2 as mean ± SEM and the p-value after an unpaired Student´s t-test complementary to Figure 5 and Figure 6.

µM	TAS2R8KD			TAS2R10KD		
	MOCK	KD	p	MOCK	KD	p
62.5	1.245 ± 0.472	-0.878 ± 0.542	0.0049	1.588 ± 0.386	-0.892 ± 0.837	0.0102
125	2.807 ± 0.540	0.366 ± 0.572	0.0033	2.930 ± 0.486	0.681 ± 0.794	0.0197
250	4.813 ± 0.587	0.933 ± 0.706	0.0001	4.268 ± 0.629	1.871 ± 0.777	0.0206
500	6.337 ± 0.880	3.185 ± 0.656	0.0059	6.818 ± 0.611	4.060 ± 0.686	0.0043
1000	6.6670 ± 1.035	3.89 ± 0.659	0.0349	7.629 ± 0.599	5.513 ± 0.580	0.0146

Table S3: List of all transport genes linked to cadmium ion transport.

**Transcript**	**Symbol**	**foldchange**	**t statistics**	**p-value**
NM_014579	SLC39A2	1.79	-1.45	0.18
BC015770	SLC39A14	1.60	-1.22	0.25
NM_021194	SLC30A1	1.45	-0.93	0.38
BC002592	SLC11A2	1.17	-0.52	0.62
**NM_001032220**	**SLC11A1**	**-1.90**	**2.83**	**0.02**
**BC110057**	**SLC39A2**	**-2.02**	**2.28**	**0.05**
NM_000578	SLC11A1	-2.00	1.72	0.12
NM_015359	SLC39A14	-1.44	1.06	0.31
NM_000617	SLC11A2	-1.17	0.66	0.53
NM_022154	SLC39A8	-1.05	0.21	0.84

Table S4: List of transition metal transport genes significantly responding to Cd^{2+}.

Transcript	Symbol	foldchange	t statistics	p-value
AK056900	SLC39A4	1.91	-2.97	0.01
AK021788	TRPM3	2.64	-3.32	0.01
NM_139002	HFE	4.41	-3.34	0.01
NM_024843	CYBRD1	2.13	-2.5	0.03
NM_000146	FTL	2.81	-2.48	0.03
NM_001063	TF	1.95	-2.38	0.04
BC112223	SLC39A10	1.69	-2.2	0.05
BC104948	NECTIN1	-3.10	3.47	0.01
NM_020372	SLC22A17	-2.10	2.86	0.02
NM_001032220	SLC11A1	-1.91	2.83	0.02
BC110057	SLC39A2	-2.03	2.28	0.05
NM_020342	SLC39A10	-1.67	2.22	0.05
